# Barriers to the care of HIV-infected children in rural Zambia: a cross-sectional analysis

**DOI:** 10.1186/1471-2334-9-169

**Published:** 2009-10-16

**Authors:** Janneke H van Dijk, Catherine G Sutcliffe, Bornface Munsanje, Francis Hamangaba, Philip E Thuma, William J Moss

**Affiliations:** 1 Medical/Malaria Institute at Macha, Macha Hospital, Choma, Zambia; 2 Department of Epidemiology, Bloomberg School of Public Health, Johns Hopkins University, Baltimore, MD, USA

## Abstract

**Background:**

Successful antiretroviral treatment programs in rural sub-Saharan Africa may face different challenges than programs in urban areas. The objective of this study was to identify patient characteristics, barriers to care, and treatment responses of HIV-infected children seeking care in rural Zambia.

**Methods:**

Cross-sectional analysis of HIV-infected children seeking care at Macha Hospital in rural southern Zambia. Information was collected from caretakers and medical records.

**Results:**

192 HIV-infected children were enrolled from September 2007 through September 2008, 28% of whom were receiving antiretroviral therapy (ART) at enrollment. The median age was 3.3 years for children not receiving ART (IQR 1.8, 6.7) and 4.5 years for children receiving ART (IQR 2.7, 8.6). 91% travelled more than one hour to the clinic and 26% travelled more than 5 hours. Most participants (73%) reported difficulties accessing the clinic, including insufficient money (60%), lack of transportation (54%) and roads in poor condition (32%). The 54 children who were receiving ART at study enrollment had been on ART a median of 8.6 months (IQR: 2.7, 19.5). The median percentage of CD4^+ ^T cells was 12.4 (IQR: 9.2, 18.6) at the start of ART, and increased to 28.6 (IQR: 23.5, 36.1) at the initial study visit. However, the proportion of children who were underweight decreased only slightly, from 70% at initiation of ART to 61% at the initial study visit.

**Conclusion:**

HIV-infected children in rural southern Zambia have long travel times to access care and may have poorer weight gain on ART than children in urban areas. Despite these barriers, these children had a substantial rise in CD4^+ ^T cell counts in the first year of ART although longer follow-up may indicate these gains are not sustained.

## Background

An estimated 2 million children under the age of 15 were living with HIV infection at the end of 2007, with almost 90% residing in sub-Saharan Africa [1]. Since the World Health Organization launched the '3 by 5' campaign in 2003 [2], dramatic improvements have been made to increase access to life-prolonging treatment for children in developing countries, with the number of children in sub-Saharan Africa receiving antiretroviral therapy increasing from approximately 50,000 in 2005 to over 150,000 by the end of 2007 [3].

Despite initial reservations about the implementation of antiretroviral treatment programs in Africa [4,5], recent reports demonstrate that treatment programs for HIV-infected children in sub-Saharan Africa can achieve outcomes similar to those in North America and Europe [6]. However, as the rollout of ART continues, concerns have been raised about how equitably access to ART has been distributed within countries [7-9]. HIV care services have primarily been implemented in urban areas and have lagged behind in rural areas, where there are shortages of trained personnel and the health care system faces many challenges [10]. Barriers faced by residents in rural areas may prevent them from accessing HIV care, including lower treatment literacy [11], greater distances and travel times to clinics [3], and fewer financial resources for transportation [12,13].

To address these issues, decentralized models for health care delivery have been developed to increase access to care in several rural settings [14-18]. Initial reports from rural programs have been promising [16-20]; however, further evaluation of rural HIV care programs is needed to understand the challenges to the care and treatment of HIV-infected persons, particularly children. We evaluated barriers to the care of HIV-infected children attending an HIV clinic in rural southern Zambia, with the goal of developing strategies to optimize the care of these children.

## Methods

### Study setting and population

HIV-infected children younger than 16 years and attending the Antiretroviral Clinic at Macha Hospital in Macha, Zambia were eligible for enrollment. Macha is located in Southern Province, approximately 80 km from the nearest town of Choma. The catchment area of Macha Hospital is populated by traditional villagers living in small, scattered homesteads, with an estimated population density of 25 persons per km^2 ^(P. Thuma, unpublished data). Macha Hospital is a 208-bed hospital administered by the Zambian Brethren in Christ Church that functions within the healthcare system of the Ministry of Health. The hospital serves as a district-level referral hospital for smaller hospitals and rural health centers within an 80 km radius, serving a population of over 150,000 persons. Macha Hospital provides care to approximately 4000 HIV-infected adults and children through the Government of Zambia's antiretroviral treatment program, with additional support from the President's Emergency Plan for AIDS Relief (PEPFAR) through the non governmental organization, AidsRelief. A program to prevent maternal-to-child HIV transmission began at Macha Hospital simultaneous with the implementation of the ART clinic in 2005.

HIV-infected children are referred to the clinic from voluntary counseling and testing programs, outpatient clinics and hospitals. Since February 2008, children born to HIV-infected women are routinely tested for HIV infection at approximately 6 weeks of age, using dried blood spot samples and HIV DNA PCR performed in Lusaka, Zambia. Clinical care is provided without charge by medical doctors and clinical officers, and adherence counseling by nurses and trained counselors. Home visits are attempted for persons who fail to return for scheduled follow-up visits. Children were considered eligible for antiretroviral therapy if they had WHO stage 3 or 4 disease, or a CD4^+ ^T cell percentage of <25% for children ≤ 11 months of age, < 20% for children 12-35 months of age, or <15% for children ≥ 36 months of age. The first-line antiretroviral treatment regimen consists of two nucleoside reverse transcriptase inhibitors (lamivudine plus zidovudine or stavudine) and a non-nucleoside reverse transcriptase inhibitor (efavirenz or nevirapine).

### Study procedures

This cross-sectional analysis was conducted within the context of an observational cohort study. HIV-infected children seeking outpatient care at Macha Mission Hospital, Choma, Zambia were prospectively enrolled into an observational cohort study beginning in September 2007 after written informed consent was obtained from a parent or guardian. The caretakers of all children who were asked to participate agreed to enroll in the study. A questionnaire developed by the study team was administered to the parent or guardian and the child was examined at the initial study visit and at each follow-up visit occurring approximately every three months. Blood specimens were collected in EDTA tubes as part of routine clinical care. Information from before study enrollment was abstracted from medical records. CD4^+ ^T cell counts and percentages were measured using the Guava Easy CD4 system (Guava Technologies, Inc., Hayward, CA), and hemoglobin was measured using the ABX MICROS 60 (Hariba ABX, France). The study was approved by the Research Ethics Committee of the University of Zambia, the Ministry of Health, Republic of Zambia and the Institutional Review Board of the Johns Hopkins Bloomberg School of Public Health.

For the present analysis, information was used from the initial study visit for HIV-infected children enrolled into the cohort study between September 2007 and September 2008. Children were classified as HIV-infected if they were older than 18 months with a positive serological test, younger than 18 months with confirmed infection by PCR either prior to or within 3 months of the initial study visit, or younger than 18 months with a positive serological test and either eligible for ART based on the 2006 WHO treatment guidelines or receiving ART at the initial study visit.

### Statistical Analysis

Data were entered in duplicate using EpiInfo (Centers for Disease Control and Prevention) and analyses were conducted in SAS for Windows version 9.1 (SAS Institute Inc., Cary, NC). Proportions are reported for categorical variables and differences were tested using chi-square tests. Medians and interquartile ranges are reported for continuous variables and differences were tested using Wilcoxon rank sum tests.

Children were categorized according to their use of ART at the time of the first study visit. Children who were not on ART were further categorized by eligibility for ART, as defined by the 2006 WHO treatment guidelines [21]. If laboratory results were not available at a specified clinic visit, results were used within a 3-month period (3 months prior for children initiating ART). Weight-for-age z-scores were calculated based on the WHO growth standards [22] and children with z-scores below -2 were defined as underweight. A measure of socio-economic status (SES) was calculated based on the Demographic and Health Survey SES scale used in Zambia [23]. SES percentiles were based on the predetermined cutoffs (<25^th ^= 0-6; 26-50^th ^= 7-12; 51-75^th ^= 13-18; >75^th ^= 19-24).

## Results

### Characteristics of study children

192 HIV-infected children were enrolled, 54 (28%) of whom were receiving ART at the initial study visit. These children represent approximately 70% of all HIV-infected children with at least one clinic visit between September 2007 and September 2008. The median age was 3.5 years (IQR: 1.9, 7.4; range: 0.3, 15.6) and 47% were boys (Table 1). The majority of children were cared for by a parent (74%), although 35% had lost one or both parents, and the majority of parents (96%) reported signs and symptoms of disease at the time of the initial clinic visit. Most children whose primary caregiver was not a parent were cared for by other family members, primarily a grandparent. The median age of the primary caregiver was 35.8 years (IQR: 30.3, 43.6) and their educational status was low, with 61% of caregivers achieving at most a primary level of education, and only 52% of these caregivers completed grade 7. The socioeconomic status of the households also was low, with 70% of children living below the 25^th ^percentile. Children receiving ART at the initial study visit were older (median age: 4.5 vs. 3.3 years) and were more likely to be male (67% vs. 40%), but were otherwise similar to children who were not receiving ART at the initial study visit. The use of antiretrovirals to prevent mother-to-child transmission in the study population was uncommon; seven children (4%), none of whom were receiving ART, received antiretrovirals in the perinatal period as confirmed by the medical record (n = 6) or self-report (n = 1).

**Table 1:** Characteristics of HIV-infected children in the ART clinic in Macha, Zambia (2007 to 2008)

	Total (n = 192)	Not on ART (n = 138)	On ART (n = 54)
***Child***			

**Median age in years (IQR)^a^**	3.5 (1.9, 7.4)	3.3 (1.8, 6.7)	4.5 (2.7, 8.6)
**Male (%)^a^**	91 (47)	55 (40)	36 (67)

***Parents***			

**Status (%)**			
Both alive	121 (65)	94 (69)	27 (54)
Mother deceased	26 (14)	20 (15)	6 (12)
Father deceased	19 (10)	11 (8)	8 (16)
Both deceased	20 (11)	11 (8)	9 (18)

***Primary Caregiver***			

**Relationship to child (%)**			
Mother/father	139 (74)	104 (76)	35 (70)
Grandparent	28 (15)	17 (12)	11 (22)
Aunt/uncle	14 (7)	11 (8)	3 (6)
Sibling	3 (2)	2 (1)	1 (2)
Other	3 (2)	3 (2)	0 (0)
**Median age (IQR)^b^**	35.8 (30.3, 43.6)	35.5 (29.2, 43.6)	38.0 (33.3, 43.5)
**Education (%)^b^**			
None	8 (5)	5 (4)	3 (7)
Primary	98 (56)	75 (58)	23 (52)
Secondary	64 (37)	46 (35)	18 (41)
Higher	4 (2)	4 (3)	0 (0)
**Household SES (%)**			
≤25^th ^percentile	131 (70)	97 (71)	34 (68)
26-50^th ^percentile	48 (26)	33 (24)	15 (30)
51-75^th ^percentile	7 (4)	6 (4)	1 (2)
76-100^th ^percentile	1 (1)	1 (1)	0 (0)

***Travel to ART clinic***			

**Median distance in km** **IQR)**	28 (18-45)	28 (17-45)	30 (18-42)

ART = antiretroviral therapy; IQR = interquartile range; SES = socioeconomic status

^a^p < 0.05 for difference between HIV-infected children on ART and not yet on ART

^b^excluding respondents who are not the child's primary caregiver (n = 13)

### Disclosure of HIV-infection status

As the study population was young, few children (3%) were reported to be aware of their HIV infection status. Among older children, only 5 (17%) of 29 children older than 10 years were aware of their status, with significantly more children receiving ART aware of their status (33% vs. 6%; *P *= 0.05). Of those children who were not informed of their HIV infection status, 83% were reported to be aware that they were sick (88% on ART vs. 81% not on ART; *P *= 0.70). Caregivers were asked to provide reasons for non-disclosure, and the primary reason (88%) was because they felt the child was too young to know. Forty-two percent of caregivers also reported that they had not disclosed the child's status because they were afraid to tell the child, 21% because they did not know how to tell the child, and 17% because they felt that it was not good for the child to know their HIV infection status.

Among all caregivers, 97% reported that someone other than themselves or the child was aware of the child's HIV infection status, with 47% reporting that only other family members had been told, 49% reporting that family members as well as others in the community (mostly neighbors) had been told, and 1% reporting that only other non-family members had been told. Significantly more caregivers of children on ART had disclosed to both family members and others in the community compared to children not receiving treatment (86% vs. 36%, *P *< 0.0001). Few caregivers (2%) reported being fearful of others finding out about their child's HIV infection status, or felt that they (3%) or their children (6%) were stigmatized because of their child's status. Significantly more caregivers of children on ART reported that they (9% vs. 1%, *P *= 0.004) or their child (16% vs. 2%, *P *= 0.0008) had been stigmatized. Among caregivers who reported feeling stigmatized, their children primarily experienced rejection by family (60%) and friends (50%), while the caregivers experienced rejection by their family (60%) and community (60%).

### Clinic access

**Figure 1:**
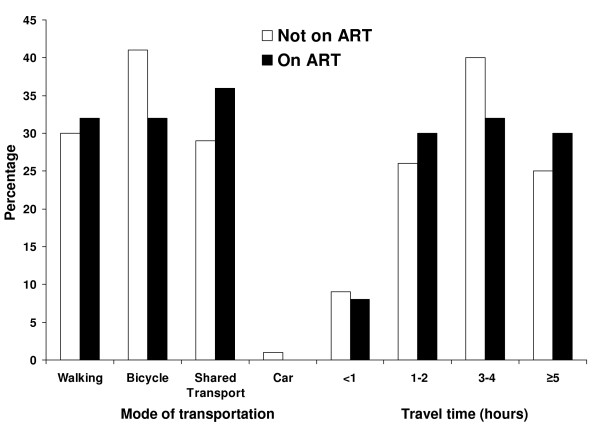
**Method of transportation and travel time to the ART clinic among HIV-infected children**. Note: Modes of transportation are not mutually exclusive.

Children and their caregivers travelled an estimated median distance of 28 km (IQR: 18, 45) to attend the clinic. The majority of participants used a single mode of transportation, including walking (30%), cycling (39%) or public transportation (31%). The majority (91%) of participants travelled more than one hour to the clinic, with 26% travelling more than 5 hours (Figure 1). For participants who used motorized vehicles, 53% spent more than 20,000 ZMK (approximately 5 USD at that time) of their own (54%) or their family's (46%) money for transportation. No significant differences were found in mode, time or cost of transportation between children who were and were not receiving ART at study enrollment.

**Figure 2:**
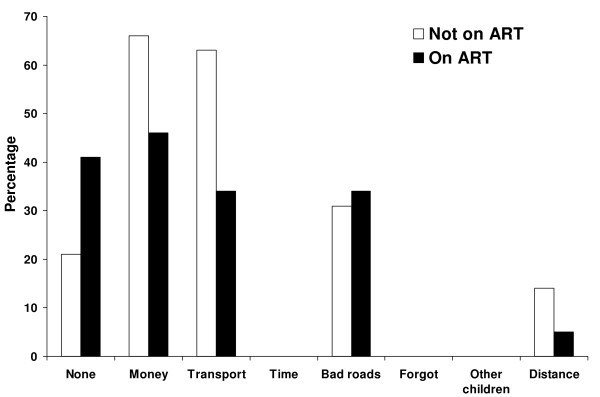
**Problems experienced by HIV-infected children and their caregivers in travelling to the ART clinic in Macha, Zambia**.

Seventy-three percent of participants reported ever experiencing problems accessing the clinic (Figure 2), primarily due to lack of money (60%), transportation (54%), and because roads were in poor condition (32%). No caretakers reported problems attending the clinic due to lack of time, forgetting about appointments or inability to find childcare. Caretakers tended to report more problems accessing the clinic during the rainy season (78%) from mid-November to mid-April than during the dry season (67%, *P *= 0.2). Significantly more children not receiving ART reported problems attending the clinic than children receiving ART (79% vs. 59%, *P *= 0.01).

### Characteristics of children not receiving ART at the initial study visit

138 HIV-infected children were not receiving ART at study enrollment (Table 2). These children had been followed in the clinic a median of 3.2 months before study enrollment (IQR: 0.5, 10.1). The median percentage of CD4^+ ^T cells was 20.7 (IQR: 16.0, 30.0), but 37% of children were severely immunosuppressed. In addition, 54% of children were classified as WHO stage 3 or 4 at the initial study visit. In this group, 79 (60%) children were eligible at the initial study visit to start treatment based on the 2006 WHO treatment guidelines [21]. These children had been followed in the clinic a median of 2.8 months (IQR: 0.4, 9.0), and for 14% of children this visit represented their first visit in the clinic. Children eligible for treatment were significantly younger (2.3 vs. 3.6), had lower median WAZ (-2.4 vs. -2.1), and, as expected, had a lower median percentage of CD4^+ ^T cells (17.4 vs. 29.6) and higher stage of disease progression (78% classified as WHO stage 3 or 4 vs. 0%). Based upon detailed review of the medical records of those children deemed eligible for ART at the initial study visit and who had been followed at the clinic a sufficient length of time to have a potential delay in initiation of ART, the most common reasons for a potential delay were loss to follow-up (10 children), concurrent tuberculosis (9 children) and need for adherence counseling (3 children).

**Table 2:** Characteristics at study enrollment of HIV-infected children not receiving ART

	Total (n = 138)	Children not eligible for ART^c ^(n = 53)	Children eligible for ART at study enrollment (n = 79)
**Median age (yrs) (IQR)^a^**	3.3 (1.8, 6.7)	3.6 (2.5, 6.7)	2.3 (1.3, 6.7)
**Male (%)**	55 (40)	20 (38)	34 (43)
**Length of time (months) since enrolling in ART clinic**	3.2 (0.5, 10.1)	3.8 (0.2, 11.7)	2.8 (0.4, 9.0)
**Median WAZ (IQR) (n = 120)^a,b^**	-2.2 (-3.2, -1.3)	-2.1 (-2.7, -1.0)	-2.4 (-3.5,-1.6)
WAZ <-2	66 (55)	25 (53)	41 (59)
WAZ ≥-2	54 (45)	22 (47)	28 (41)
**Median CD4^+ ^T cell % (IQR) (n = 122)^a^**	20.7 (16.0, 30.0)	29.6 (23.2, 36.9)	17.4 (12.1, 21.1)
**Median CD4^+ ^T cell count (IQR) (n = 126)^a^**	815 (455, 1372)	980 (578, 1434)	735 (377, 1287)
**Severe immunodeficiency^a,d^**	47 (37)	0 (0.0)	47 (64)
**Median total lymphocyte count (IQR) (n = 73)**	4266 (2238, 7025)	3289 (2179, 4847)	4464 (2328, 7236)
**Median hemoglobin (IQR) (n = 110)^a^**	10.5 (9.2,11.6)	11.3 (9.8,11.7)	10.1 (8.8, 11.3)
Hemoglobin <8 gm/dL	6 (5)	1 (3)	5 (7)
Hemoglobin ≥8 gm/dL	104 (95)	36 (97)	68 (93)
**WHO stage (n = 101)^a^**			
1	17 (16)	11 (34)	5 (7)
2	33 (32)	21 (66)	10 (14)
3	38 (37)	0 (0)	38 (55)
4	16 (15)	0 (0)	16 (23)

ART = antiretroviral therapy; IQR = interquartile range; WAZ = weight-for-age Z score; WHO = World Health Organization

^a ^p < 0.05 for difference between children eligible and ineligible for ART at study enrollment

^b ^for children <10 yrs old

^c ^eligibility for ART determined based on the 2006 WHO treatment guidelines; 6 children could not be classified as WHO stage and laboratory results were not available at baseline

^d ^severe immunodeficiency defined by age according to WHO guidelines

### Response to therapy among children receiving ART at the initial study visit

**Figure 3:**
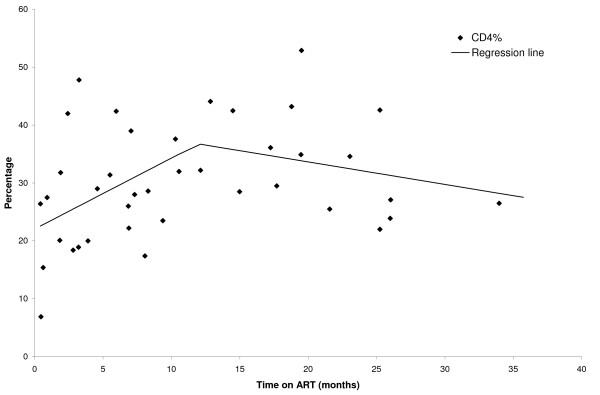
**Percentage of CD4^+ ^T cells after ART initiation for HIV infected children in Macha, Zambia**.

54 children were receiving ART at study enrollment (Table 3). These children had been followed in the clinic a median of 13.6 months (IQR: 6.4, 23.2) and had been on ART a median of 8.6 months (IQR: 2.7, 19.5). The median percentage of CD4^+ ^T cells was 12.4 (IQR: 9.2, 18.6) at the start of ART and increased to 28.6 (IQR: 23.5, 36.1) at the initial study visit. The median increase in percentage of CD4^+ ^T cells from ART initiation to the initial study visit was 18.1 (IQR: 6.0, 26.8). The percentage of CD4^+ ^T cells increased with the duration of ART use (Figure 3). The proportion of children with a percentage of CD4^+ ^T cells greater than 25% increased from 64% among those who had received ART for less than 12 months (n = 22) to 83% among those who had received ART for at least 12 months (n = 18). Children were not consistently classified according to WHO clinical stage; however, among those with WHO staging available (28 at ART initiation and 34 at the initial study visit) there was a decrease in the proportion of children in stage 3 or 4 from 89% at ART initiation to 65% at the initial study visit. However, the proportion of children who were underweight decreased only slightly, from 70% at ART initiation to 60.5% at the initial study visit for 37 children with available data. WAZ scores increased a median of 0.24 (IQR: -0.57, 0.84) from initiation of ART to the initial study visit.

**Table 3:** Characteristics of children receiving ART (n = 54)

	At clinic enrollment	At ART initiation^a^	At study enrollment
**Median age (yrs) (IQR)**	3.6 (1.5, 7.7)	3.8 (2.0, 8.0)	4.5 (2.7, 8.6)
**Median WAZ (IQR)^b^**	-2.7 (-3.5, -1.5)	-2.5 (-3.9, -1.8)	-2.4 (-3.2, -1.5)
WAZ <-2	27 (61.4)	28 (70.0)	26 (60.5)
WAZ ≥-2	17 (38.6)	12 (30.0)	17 (39.5)
**Median CD4^+ ^T cell % (IQR)**	14.0 (9.2, 19.0)	12.4 (9.2, 18.6)	28.6 (23.5, 36.1)
**Median CD4^+ ^T cell count (IQR)**	505 (281, 985)	368 (219, 559)	973 (466, 1522)
Severe immunodeficiency^c^	16 (50)	24 (63)	2 (5)
**Median total lymphocyte count (IQR)**	3194 (1948, 5351)	2747 (1634, 5351)	3372 (1862, 5276)
**Median hemoglobin (IQR)**	10.6 (10.0, 11.7)	10.8 (10.0, 12.0)	11.9 (10.6, 12.3)
Hemoglobin <8 gm/dL	1 (2.0)	0 (0)	4 (8.9)
Hemoglobin ≥8 gm/dL	49 (98.0)	36 (100.0)	41 (91.1)
**WHO stage**			
1	2 (5.1)	2 (7.1)	6 (17.7)
2	9 (23.1)	1 (3.6)	6 (17.7)
3	22 (56.4)	20 (71.4)	20 (58.8)
4	6 (15.4)	5 (17.9)	2 (5.9)
**ART regimen**			
AZT/3TC/EFV	---	12 (26.1)	11 (42.3)
AZT/3TC/NVP	---	11 (23.9)	7 (26.9)
d4T/3TC/EFV	---	5 (10.8)	2 (7.7)
d4T/3TC/NVP	---	18 (39.1)	6 (23.1)

ART = antiretroviral therapy; IQR = interquartile range; WAZ = weight-for-age Z score; WHO = World Health Organization; AZT = zidovudine; 3TC = lamivudine; EFV = efavirenz; NVP = nevirapine; d4T = stavudine

^a^7 children transferred to Macha and had already initiated ART

^b^for children <10 yrs old

^c^severe immunodeficiency defined by age according to WHO guidelines

### ART regimens and adherence

ART regimens at treatment initiation and the initial study visit consisted of a combination of 2 NRTIs and 1 NNRTI. Side effects were rarely reported, with only two children reporting a rash and one child reporting headaches after treatment initiation. A median of two adults (IQR: 2-3) were involved in supervising the administration of ART. The primary person responsible for ART administration was the child's mother (56%). Sixty-one percent of children were living in households where at least one other person, primarily a parent, also was receiving ART. Among respondents, 22% of 41 reported problems with adherence. Among those who did, the primary reasons for a child missing a dose were forgetting (n = 2), the pills being either too numerous (n = 1) or too large to swallow (n = 1), the child refusing (n = 1), side effects (n = 2), the caregiver being away (n = 1), and finishing the supply of drugs prior to the next clinic appointment (n = 1).

## Discussion

Most assessments of the care of HIV-infected children in sub-Saharan Africa have been from urban sites, often from established research programs [6]. Our observations highlight barriers to the care of HIV-infected children unique to rural settings, specifically long travel times and lack of transportation, but are encouraging in that age at clinic enrollment and immunologic outcomes in the first year of treatment did not differ substantially from published reports on the care of HIV-infected children in urban sub-Saharan Africa. These findings suggest that the barriers to the care of HIV-infected children in rural settings do not pose insurmountable obstacles to desirable treatment outcomes.

The median age of HIV-infected children receiving care in rural southern Zambia was younger than commonly reported (4.5 years for children receiving ART and 3.3 years for children not receiving ART). Among studies reporting the effectiveness of pediatric ART in sub-Saharan Africa, 73% of 26 studies reported a median or mean age of five years or older at initiation of ART [6]. In Lusaka, Zambia, the median age at which HIV-infected children began care was 5.4 years (IQR: 1.9, 9.5) [24]. Thus, compared to this urban Zambian pediatric cohort, children accessing care in this rural setting do not appear to face additional delays; however, the number of children undiagnosed or not accessing care is not known. There is urgent need to diagnose HIV infection and initiate ART within the first year of life given the high mortality rate in early childhood [25] and the demonstrated benefits of initiating treatment in early infancy [26]. In rural Zambia, as elsewhere in sub-Saharan Africa, children accessing care are a subset of HIV-infected children with slower disease progression. At Macha Hospital, infant diagnosis of HIV infection using dried blood spots and PCR was introduced in February 2008, and is likely to reduce the age at which HIV-infected children enter care.

One of the major barriers to care in rural sub-Saharan Africa is access to health facilities. In rural southern Zambia, more than 90% of HIV-infected children travelled more than one hour to the clinic and more than one quarter travelled more than five hours. Lack of transportation, insufficient financial resources and poor roads were commonly cited obstacles to accessing the clinic, particularly during the rainy season. Such obstacles are unlikely to be major barriers to care in urban settings and could result in suboptimal treatment outcomes for HIV-infected children residing in rural areas. Significantly more children not on ART reported problems accessing the clinic but the reasons are unknown. It may be that caregivers who have committed to providing ART for their child perceive fewer problems, as the journey to and from the clinic has become routine.

The majority of children (78%) were not receiving ART at the time of study entry. Although these children were followed for only a median of 3.2 months, some were deemed eligible for ART at study entry. Over a third were severely immunosuppressed and slightly more than half were classified as WHO stage 3 or 4 at the initial study visit. Consequently, many of these children were eligible for ART at the study visit according to the WHO guidelines. However, many met criteria for WHO stage 3 because of poor nutritional status, which in this setting does not necessarily represent advanced HIV disease and is not in itself a criterion for treatment initiation. In addition, many children had not been followed in the clinic long enough to fulfill the requirements for treatment initiation, including eligibility and adherence visits. For children who had been in the clinic a sufficient time, delayed initiation of ART could have resulted from biomedical, social or health care-associated factors. In a study conducted in Pretoria, South Africa, reasons for delayed initiation of ART among 147 eligible, HIV-infected children included: tuberculosis co-infection (26%); inadequate clinic staffing (20%); social problems, including lack of transportation, absence of legal guardian and denial (17%); and inaccurate clinical or immunological staging (14%) [27]. In this study, delays in initiation of ART most frequently resulted from loss to follow-up or concurrent tuberculosis. Given the advanced clinical and immunological stages at which children entered the clinic, delays in ART initiation could be detrimental, leading to higher pre-ART mortality [28], more rapid disease progression [26], or poorer response to treatment.

In rural Zambia, three quarters of the HIV-infected children seeking care had a parent as the primary caregiver, although one third had lost at least one parent. Children who are orphans [29] and children whose primary caregiver is not a parent [30] may be at increased risk for non-adherence to the antiretroviral regimen. Few children, even among the older age groups, were reported to be aware of their HIV-infection status, primarily because the caregivers thought the child too young to be told the diagnosis or they were afraid to tell the child. However, this finding must be interpreted with caution as this information was reported by the caregiver rather than the child and may be an underestimate of the children's true knowledge of their status.

Despite these barriers, the 54 children receiving ART at study entry achieved good immunological responses, with the percentage of CD4^+ ^T cells increasing a median of 17.6% after a median of 8.1 months of therapy. This compares favorably with 28 published studies of the immunological response to ART among HIV-infected children in sub-Saharan Africa residing largely in urban settings, in which the median gain in percentage of CD4^+ ^T cells was 7-14% at 6-8 months and 10-16% at 12-15 months of therapy [6]. In contrast, weight gain among HIV-infected children receiving ART in rural Zambia did not achieve the levels reported elsewhere in sub-Saharan Africa, with WAZ scores increasing a median of 0.24 after receiving ART a median of 8.6 months. Among 17 published studies that reported nutritional outcomes, weight-for-age Z scores improved by 1 SD after twelve months of ART [6]. Similarly, among 749 HIV-infected Ugandan children who received ART for a mean of 6 months, the mean weight-for-age Z score increased from -3.2 to -2.1 over the study period [31]. HIV-infected children receiving ART in rural sub-Saharan Africa may have poorer weight gain because of lower dietary intake than children residing in urban areas.

## Conclusion

HIV-infected children in rural southern Zambia have long travel times to access care and may have poorer weight gain on ART than children in urban areas. Despite these barriers, children in rural Zambia had a substantial rise in CD4^+ ^T cell counts in the first year of ART although longer follow-up may indicate these gains are not sustained. Developing strategies to improve access to care and nutrition will be necessary to ensure optimal, long-term treatment outcomes for HIV-infected children residing in rural sub-Saharan Africa.

## List of Abbreviations

3TC: lamivudine; ART: antiretroviral therapy; AZT: zidovudine; d4T: stavudine; EFV: efavirenz; IQR: interquartile range; NVP: nevirapine; SES: socio-economic status; USD: United States dollar; WAZ: weight-for-age Z-score; WHO: World Health Organization; ZMK: Zambian Kwacha

## Competing interests

The authors declare that they have no competing interests.

## Authors' contributions

JHvD conceived of the study, supervised the implementation of the study in Zambia, and participated in the writing of the manuscript. CGS performed the data analysis and participated in the writing of the manuscript. BM was responsible for study recruitment and implementation, and reviewed the final manuscript. FH was responsible for study recruitment and implementation, and reviewed the final manuscript. PET supervised the implementation of the study in Zambia and participated in the writing of the manuscript. WJM conceived of the study, supervised the implementation of the study in the US and participated in the writing of the manuscript. All authors have read and approved the final manuscript.

## Pre-publication history

The pre-publication history for this paper can be accessed here:

http://www.biomedcentral.com/1471-2334/9/169/prepub
